# Early distant relapse after optimal local control in locally advanced rectal cancer

**DOI:** 10.1186/1477-7800-5-18

**Published:** 2008-07-14

**Authors:** Javier Gallego-Plazas, Francisco Menarguez-Pina, Natividad Martinez-Banaclocha, Vanesa Pons-Sanz, Fernando Mingol-Navarro, Jose A Ruiz-Macia, Sonia Macia-Escalante

**Affiliations:** 1 Servicio de Oncología Médica, Hospital General Universitario de Elche, Elche, Alicante. Spain; 2 Servicios de Cirugía, Hospital Vega Baja, Orihuela, Alicante, Spain; 3 y Cirugía, Hospital General Universitario de Elche, Elche, Alicante, Spain; 4 y Anatomía Patológica, Hospital Vega Baja, Orihuela, Alicante, Spain

## Abstract

We present a case of locally advanced rectal cancer with initial optimal local control after neoadjuvant concurrent chemoradiotherapy followed by surgery; early liver recurrence then occurred and was treated again with curative intent with neoadjuvant combination chemotherapy followed by liver surgery. We reflect on this difficult problem and discuss relevant topics to this case report.

## Clinical case

A male of 56 years of age with clinical history of hyperuricemia and gout was hospitalised because of rectal bleeding. His symptoms had started two months prior, and he had been diagnosed with haemorrhoids.

**Figure 1:**
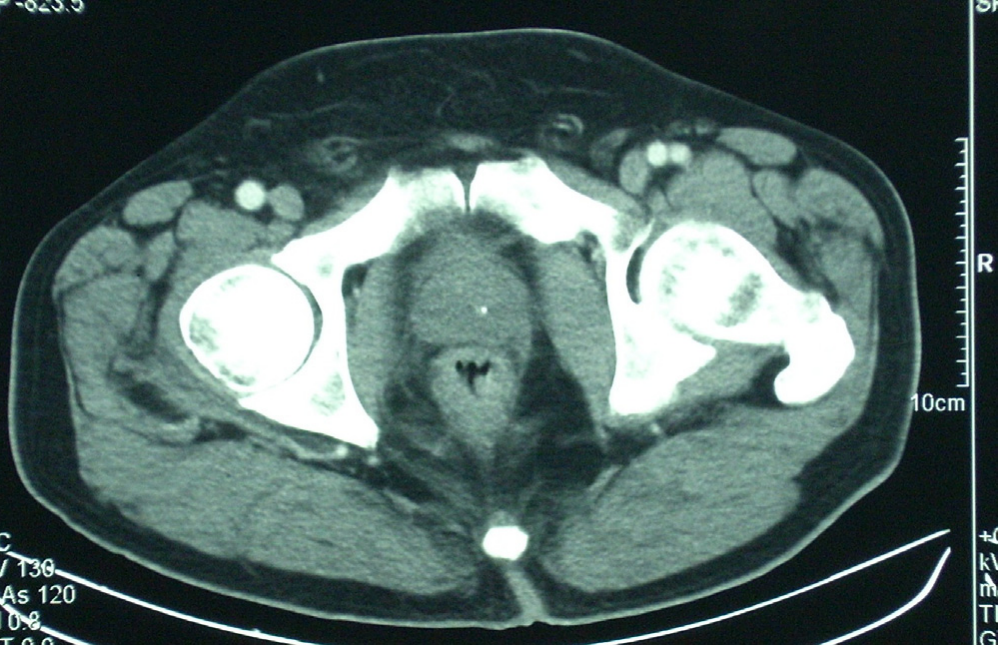
CT at diagnosis showing an eccentric thickening of the rectal wall, compatible with a rectal cancer with no lymph node or visceral involvement.

**Figure 2:**
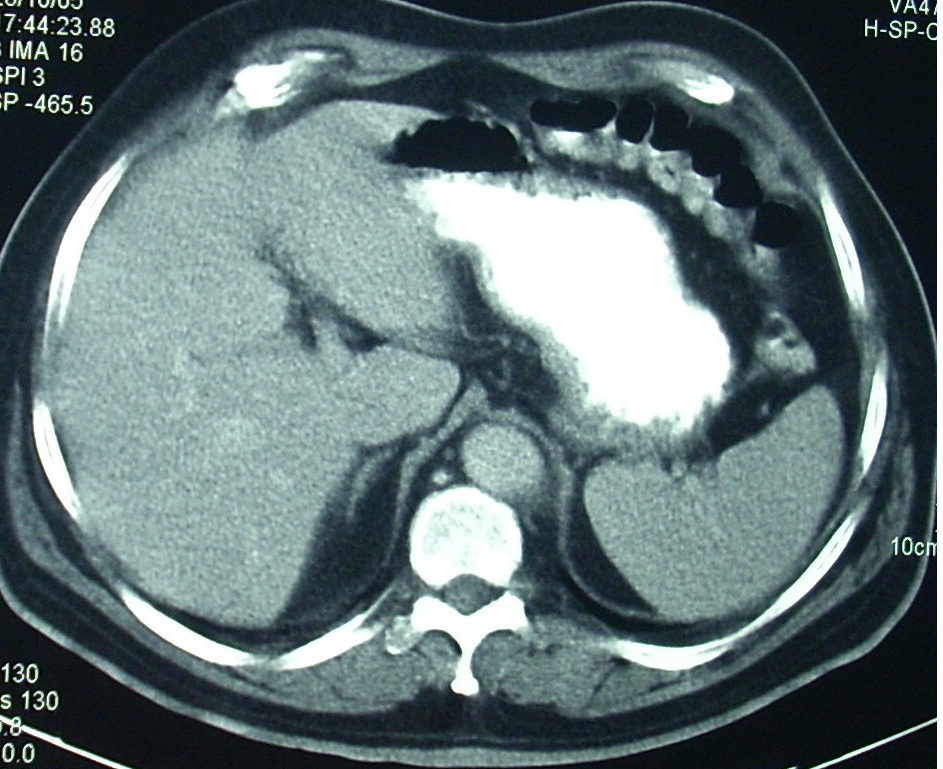
CT at initial diagnosis showing absence of liver involvement.

On admission, he had mild anemia. Blood chemistry and coagulation were normal. A full colonoscopy was performed, which detected a 5 cm long, non stenosing rectal tumour, starting after the dentate line, in addition to a sigmoid polyp. Biopsies revealed a rectal adenocarcinoma and a non dysplastic adenomatous polyp in sigmoid colon. Staging studies were completed with tumour markers (CEA and CA 19.9) measurement, echoendoscopy and a thoracic-abdominal-pelvic CT. Tumour markers values were within normal range, echoendoscopy showed a 6 cm long uT3N0 rectal cancer, and CT detected an eccentric thickening of the rectal wall, compatible with a rectal cancer with no lymph node or visceral involvement. Final diagnosis was a rectal adenocarcinoma located in the middle-inferior thirds, clinical stage T3 N0 M0 (Figures. 1, 2).

**Figure 3:**
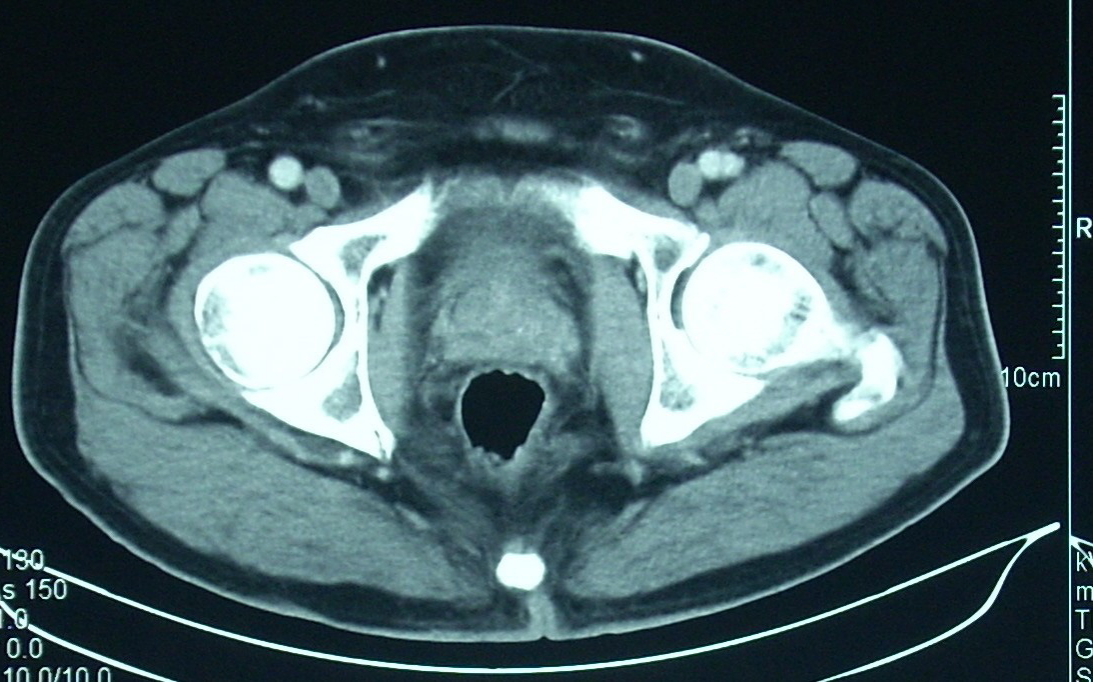
CT reassessment of primary rectal tumour after neoadjuvant treatment.

His clinical case was discussed shortly after in our Digestive Tumours Commitee, and neoadjuvant combined chemoradiotherapy followed by surgery were planned. The patient received capecitabine 900 mg/m2/12h d1-5/7d concurrent with radiotherapy, 45 Gy (180 cGy/d) [1]. Treatment was generally well tolerated, with moderate cystitis and mild epithelitis as major adverse effects. Reassessment after neoadjuvant treatment showed neither blood analysis abnormalities, nor CT suspicion of residual disease (Figure. 3).

**Figure 4:**
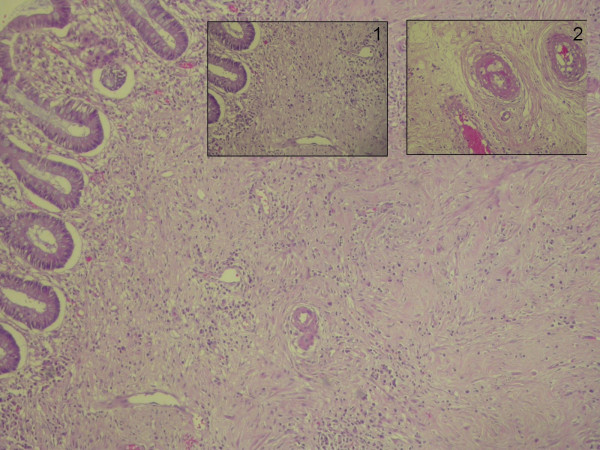
Submucosal fibrotic scarring. Detail of vertical vessels (1). Detail of radiotherapy induced obliterant vasculitis with foamy histiocytes in intimae (2).

Fifty-three days after completing chemoradiotherapy patient underwent surgery. An abdominoperineal (AP) resection, including with mesorectal excision (TME), was performed with no surgical complications. Subsequent pathological analysis revealed a complete pathological response (TRG 1) [2] (Figure. 4); with none out of four isolated lymph nodes involved; mesorectal excision was complete and the circumferential margin was greater than 1 cm.

Five weeks after surgery the patient was again referred to Clinical Oncology, were, once we had confirmed the absence of disease by blood analysis and imaging, adjuvant treatment was planned. Adjuvant fluorouracil-based chemotherapy was then administered in order to complete a total of six months neoadjuvant and adjuvant treatment [3,4]. Thoracic-abdominal-pelvic CT performed shortly after completing adjuvant chemotherapy, and this showed only surgical changes; colonoscopy through the end colostomy was normal, and blood analysis, including CEA and CA 19.9 levels, were also normal. The patient entered into our three-monthly periodic follow-up program [5].

**Figure 5:**
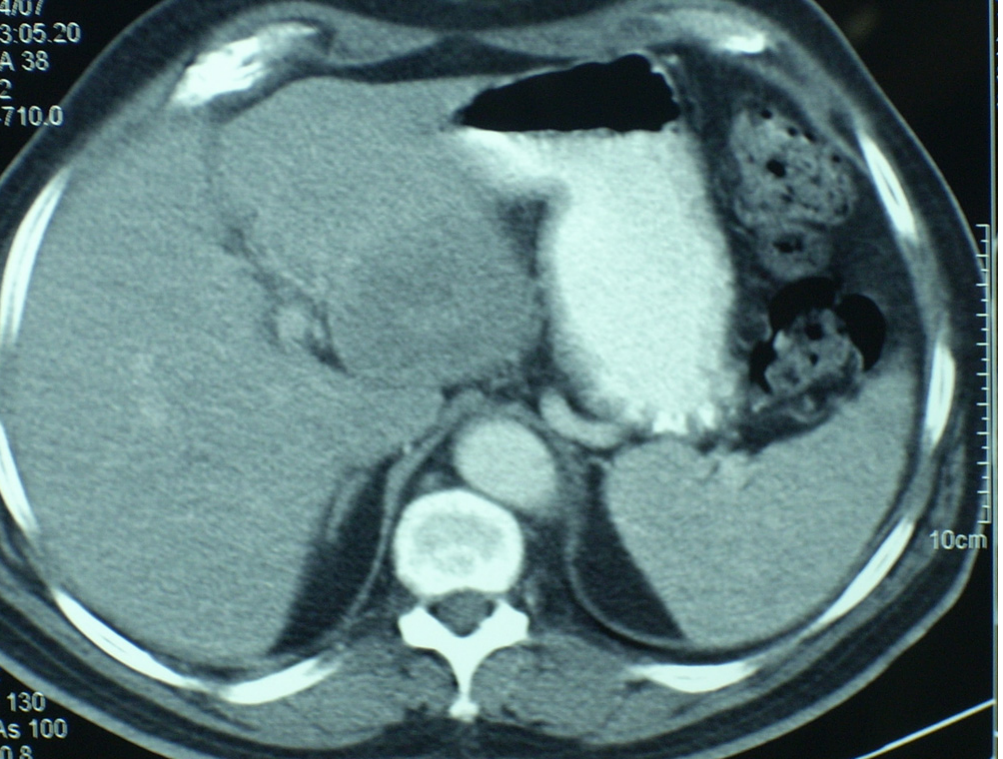
Left lobe liver metastatic lesion of size 6 cms. in thoracic-abdominal-pelvic CT performed in February 2007.

**Figure 6:**
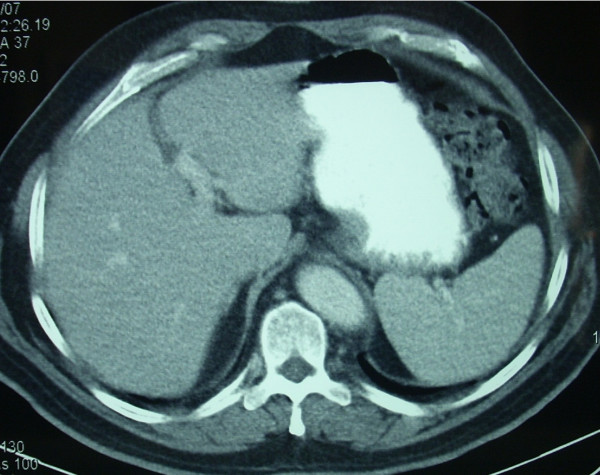
Clinical complete response assesed in thoracic-abdoninal-pelvic CT performed in May 2007, after neoadjuvant systemic chemotherapy.

**Figure 7:**
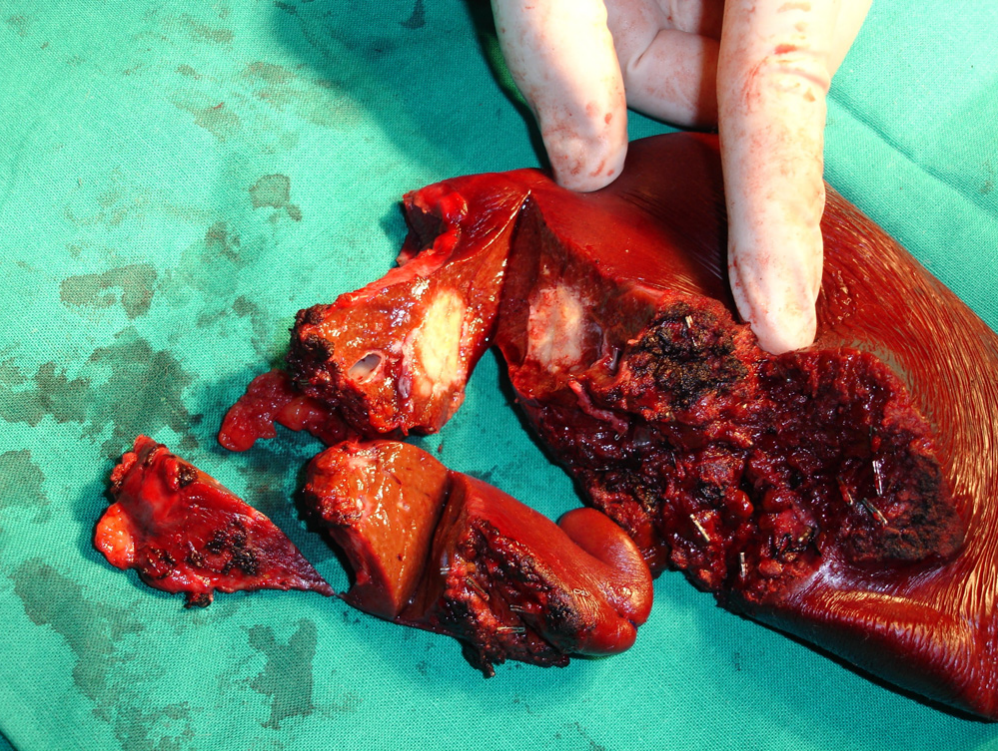
Trisegmentectomy (segments 1,2, and 3) of the liver, showing residual disease after neoadjuvant chemotherapy.

Eighteen months after initial diagnosis, the patient was asymptomatic. Programmed review detected increased CEA value (25 u/ml, normal value < 10) associated with a left lobe liver metastatic lesion of size 6 cms. in thoracic-abdominal-pelvic CT (Figure. 5). PET scan performed showed no other metastatic sites [6]. The patient received neoadjuvant chemotherapy with combination of intravenous fluorouracil c.i., oxaliplatin [7]. After completing four cycles, revaluation tests showed normalisation of CEA levels and complete disappearance of the liver lesion (Figure. 6). Patient was then referred to surgery. He subsequently had a liver trisegmentectomy, involving segments 1, 2 and 3 (Figure. 7). Pathological analysis revealed a 1 cm residual focus of metastatic non-expressing EGFR colorectal adenocarcinoma. Recently, six weeks after liver surgery, and in the absence of residual disease, patient has started adjuvant chemotherapy with FOLFOX-4, planned to last for four months.

## Discussion

Concurrent preoperative chemoradiotherapy has proven superior to other treatment strategies against locally advanced rectal cancer. Neoadjuvant combined treatment reduces local relapse compared to adjuvant combined treatment [8] and exclusive preoperative radiotherapy [9,10]. Nevertheless, this benefit not always means an increase in terms of overall survival. Inadequate doses of chemotherapy, with radiosensitizer but without systemic effect, and sub-optimal chemotherapy strategies may both well explain this paradox. The clinical case previously commented is an example of what may occur in early stage rectal cancer patients. Far from presenting local relapse, distant metastases may occur, and is being seen with increasing frequency.

By combined neoadjuvant chemoradiotherapy, followed by surgery including total mesorectal excision, it is clear that very good local control of rectal cancer can be achieved, with five-year local relapse rates of 6–8% [8-10]. This very good local control rate may be optimized when effectiveness of neoadjuvant treatment is demonstrated after surgery of regressive disease, and adjuvant chemotherapy with a similar regimen to that previously used in the neoadjuvant setting is completed [4]. The challenge on the horizon is then to reduce distant relapse, in order to prolong overall survival. The best way to accomplish this goal might be to divide preoperative treatment into two steps: neoadjuvant combined chemotherapy followed by neoadjuvant concurrent chemoradiotherapy. First step would include only combined chemotherapy, so that optimal doses and regimens with demonstrated systemic effect could be safely administered, within an attempt to control micrometastatic systemic disease. In a second stage treatment would focus on local control by concurrent administration of radiotherapy and chemotherapy, this time using adjusted doses in order to achieve synergistic effect avoiding excessive toxicity. This approach has already been partially succesfully tested in phase II clinical trials [11], and is yet to be confirmed in on course phase III clinical trials.

Fortunately, continuous investigation-based advances have made potentially curative treatment strategies available to patients, as the one here reported, even in the case of distant relapse, and when no evidence of extrahepatic disease is found [6]. Combination chemotherapy with fluroropyrimidines (fluorouracil or capecitabine), oxaliplatin, or irinotecan, and-more recently-bevacizumab or cetuximab, has improved response rates, progression-free survival and, in three trials, overall survival [12-15]. Combinations of three of these drugs have achieved best response rates so far in metastatic colorectal cancer, and so should be advised in the neoadjuvant setting of potentially resectable liver only metastatic disease [12-15]. Recent results favour, in case of resectable or potentially resectable liver only metastatic disease, combination systemic perioperative treatment in order to achieve best overall survival [7,16,17]. Eventhough complete clinical response to neoadjuvant chemotherapy may be achieved, resection of metastatic sites, when possible, is mandatory [18].

In conclusion, although recent improvements in treatment of advanced colorectal cancer make it possible to offer certain subsets of patients potential healing even in case of relapse after early disease, future treatment strategies in locally advanced rectal cancer need to focus not only in achieving optimal local control but in avoiding distant failure.

## Authors' contributions

JG wrote the manuscript and was responsible for main decissions and assistency related to this patint, FM was responsable for rectal surgery, NM represented Clinical Oncology Department in multidisciplinary team sessions, VP was second assistant physician for this patient, FM was responsable for liver surgery, JAR was responsable for anatomopathology analysis, and SM was responsable for figures.
